# Spatiotemporal endothelial cell – pericyte association in tumors as shown by high resolution 4D intravital imaging

**DOI:** 10.1038/s41598-018-27943-8

**Published:** 2018-06-25

**Authors:** Ann L. B. Seynhaeve, Douwe Oostinga, Rien van Haperen, Hanna M. Eilken, Susanne Adams, Ralf H. Adams, Timo L. M. ten Hagen

**Affiliations:** 1 Laboratory Experimental Surgical Oncology, Department of Surgery, Erasmus MC, 3015CE Rotterdam, The Netherlands; 2 Department of Cell Biology, Erasmus MC, 3015CE Rotterdam, The Netherlands; 3 Department of Tissue Morphogenesis, Max-Planck-Institute for Molecular Biomedicine, and Faculty of Medicine, University of Münster, D-48149 Münster, Germany

## Abstract

Endothelial cells and pericytes are integral cellular components of the vasculature with distinct interactive functionalities. To study dynamic interactions between these two cells we created two transgenic animal lines. A truncated eNOS (endothelial nitric oxide synthase) construct was used as a GFP tag for endothelial cell evaluation and an inducible Cre-lox recombination, under control of the Pdgfrb (platelet derived growth factor receptor beta) promoter, was created for pericyte assessment. Also, eNOStag-GFP animals were crossed with the already established Cspg4-DsRed mice expressing DsRed fluorescent protein in pericytes. For intravital imaging we used tumors implanted in the dorsal skinfold of these transgenic animals. This setup allowed us to study time and space dependent complexities, such as distribution, morphology, motility, and association between both vascular cell types in all angiogenetic stages, without the need for additional labeling. Moreover, as fluorescence was still clearly detectable after fixation, it is possible to perform comparative histology following intravital evaluation. These transgenic mouse lines form an excellent model to capture collective and individual cellular and subcellular endothelial cell – pericyte dynamics and will help answer key questions on the cellular and molecular relationship between these two cells.

## Introduction

Blood vessels consist of an endothelial lining surrounded by perivascular cells (i.e. pericytes and vascular smooth muscle cells). Endothelial cells form the inner layer sustaining a dynamic barrier between underlying tissue and blood. Perivascular cells are wrapped around endothelial cells, provide structural support to the vessel tube and regulate vascular tone, although the complex molecular association between both cells suggests that pericytes are more than just supporting cells (for review^1,2^). While presence of pericytes in the vasculature has been documented in the past and is reviewed by Simms in 1986^3^, more intensive investigation into lineage^4,5^, function^6^, and motility^7^, especially in association with endothelial cells, is more recent as is recognition of a therapeutic target^8–11^. As pericytes express different markers and the expression profile varies between subtypes, species, tissue and pathological conditions^12–17^, it is a more challenging cell type to investigate. Angiogenesis, the formation of new blood vessels from a pre-existing vascular network, is a very dynamic biological process which involves a series of interdependent and multicellular processes. In general it starts with sprouting of endothelial cells^18,19^ from existing vessels, followed by formation of a functional tube through anastomosis^20^, pruning^21,22^ and re-introduction of perivascular cells^16,23^. Depicting angiogenesis by classical histology provides a static image and is often not sufficient for a correct interpretation of kinetics as such spatiotemporal complexity requires successive observations in a 4D (XYZ spatial + T, time dimension) intravital manner. A major technological development which improved possibilities for longitudinal cellular investigation *in vivo* is the introduction of fluorescent proteins to the genome of animals, most often mice and zebrafish^24–26^.

In this report we demonstrate the use of two transgenic mouse lines expressing fluorescent proteins in both endothelial cells and pericytes. We generated a transgenic mouse line using the eNOS (endothelial nitric oxide synthase) promoter as a tag controlling GFP expression to evaluate endothelial cells, and a line with an inducible Cre-lox recombination under control of the Pdgfrb (platelet derived growth factor receptor beta) promoter for assessment of pericytes. Another reliable marker for pericytes is Cspg4 (chondroitin sulfate proteoglycan 4) and we crossed our eNOStag-GFP mouse with the already established Cspg4-DsRed mouse line^27^. We used a tumor transplanted in the dorsal skinfold chamber as an angiogenic model in order to achieve high resolution 4D intravital imaging and evaluated spatial, temporal and morphological interactions between endothelial cells and pericytes.

## Results

### Fluorescence in endothelial cells and pericytes

**Figure 1:**
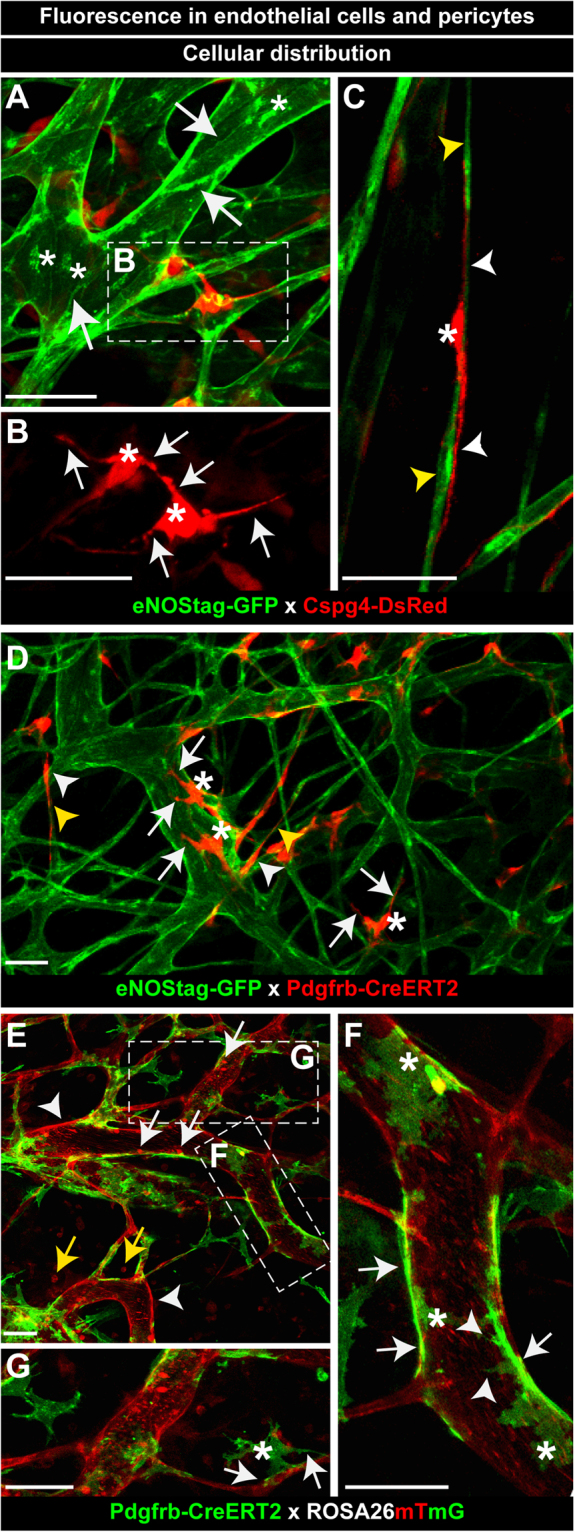
Fluorescence in endothelial cells and pericytes. (**A**–**C**) GFP Fluorescence in endothelial cells and DsRed in pericytes. (**A**) GFP fluorescence in the membrane (arrow) and golgi (asterisk) of tumor-associated endothelial cells. Cytosolic-DsRed fluorescence in Cspg4+ tumor-associated pericytes. (**B**) DsRed fluorescence in the pericyte body (asterisk) and pericyte processes (white arrow). (**C**) GFP fluorescence in long thin endothelial cells (yellow arrowhead). DsRed fluorescence in the pericyte body (asterisk) and long extended protrusions (white arrowhead). (**D**) GFP fluorescence in endothelial cell and Cre-induced cytosolic-tdTomato fluorescence in Pdgfrb+ pericytes. TdTomato fluorescence in the round pericyte body (asterisk) with short pericyte processes (arrow) and the more stretched body (white arrowhead) with longer protrusions (yellow arrowhead). (**E**–**G**) Membrane-tdTomato and Cre-induced membrane-eGFP fluorescence in Pdgfrb+ pericytes. (**E**) Tomato fluorescence in vascular cells (white arrowhead), circulating cells (white arrow) and cells in the tumor interstitium (yellow arrow). (**F**,**G**) GFP fluorescence in short (arrowhead) and long (arrow) pericyte processes and in the pericyte body (asterisk). Scale bars represent 50 µm.

To explore endothelial – pericyte association eNOStag-GFP mice were crossed with Cspg4-DsRed mice (Suppl. Fig. S1C). Both constitutive expressed fluorescent labels were clearly visible when imaged intravitally (Fig. 1A) and were homogeneously distributed throughout the tumor-associated vasculature (Suppl. Fig. S2A). Using a tumor as an angiogenic model has the advantage that in a single tumor all stages of tumor vessel development were observed: areas void of vessels (Suppl. Fig. S2A; yellow asterisk), progressing angiogenic vessels (Suppl. Fig. S2A; arrow) next to dense regions with an already established vasculature (Suppl. Fig. S2A; white asterisk) and destroyed vessels (Suppl. Fig. S2A; double white asterisk) identified by granulated cellular leftovers still fluorescent for GFP or DsRed. These leftovers are positive for TUNEL staining (Suppl. Fig. S2B; arrow) indicative of apoptosis. Established vessels showed the typical endothelial cobblestone like morphology that is also seen in 2D *in vitro* cultures using Huvecs. Expression of eNOStagged GFP was observed in the golgi (Fig. 1A; asterisk, Suppl. Fig. 2C; arrow) and cellular membrane (Fig. 1A; arrow), allowing visualization of cell-cell contacts. DsRed fluorescence in pericytes was seen in the cellular body (Fig. 1B; asterisk) and in several processes protruding from the cellular body (Fig. 1B; arrow). Also, fluorescence in long thin endothelial sprouts (Fig. 1C; yellow arrowhead) and associated Cspg4+ pericytes was detected. Pericyte cellular bodies (Fig. 1C; asterisk) were thin with longer processes protruding from opposite sides of the body (Fig. 1C; white arrowhead). Secondly, eNOStag-GFP mice were crossed with an inducible Pdgfrb(BAC)-CreERT2xROSA-tdTomato line, expressing tomato fluorescence in Pdgfrb+ cells after tamoxifen administration (Suppl. Fig. S1E). Distribution of labeled Pdgfrb+ pericytes was heterogeneously dispersed throughout the tumor, most likely as a result of more mosaic recombination, referred to as hotspots (Suppl. Fig. S2D; asterisk). Fluorescent expression and morphology of Pdgfrb+ cells in this mouse line (Fig. 1D) were similar to Cspg4+ cells. TdTomato fluorescence was seen throughout the cellular body (Fig. 1D; asterisk) and in multiple short processes (Fig. 1D; arrow). Also, when associated with stretched endothelial cells cellular bodies of Pdgfrb+ pericytes were thinner (Fig. 1D; white arrowhead) with long extensions (Fig. 1D; yellow arrowhead). There were no indications that Pdgfrb+ cells were positive for the anti-FAP-1 fibroblast marker (Suppl. Fig. S2E). Thirdly, Pdgfrb(BAC)-CreERT2 mice were crossed with a ROSA-mTmG receptor line (Suppl. Fig. S1D). ROSA-mTmG mice have a membrane-localized two-color fluorescence Cre reporter allele. The line expresses membrane-tdTomato fluorescence in widespread cells and membrane-eGFP in Cre recombinase expressing cells. The red fluorescence was expressed by the stromal part of the tumor; in the membrane of vascular cells (Fig. 1E; white arrowhead), circulating cells (Fig. 1E; white arrow, Suppl. Video S1) and infiltrated blood cells (Fig. 1E; yellow arrow). Fluorescence of eGFP was observed in Pdgfrb+ cells after Cre-induced recombination using tamoxifen. Membrane-localized eGFP fluorescence was less condense and had a more speckled appearance compared to DsRed and tdTomato fluorescence that is known to be soluble in the cytosol^27^. Due to the membrane-anchoring of the GFP protein, even small cellular protrusions were labeled and, accordingly, GFP+ cells appeared star-shaped (Fig. 1F; asterisk) with long extensions (Fig. 1F; arrow) and shorter processes (Fig. 1F; arrowhead). These star-shaped cells (Fig. 1G; asterisk) also connected to endothelial partners only through branches (Fig. 1G; arrow). In addition, another well-known model to study sprouting angiogenesis and vessel maturation is the developing retina^28^ and animals described in this paper can be used to study retinal angiogenesis as fluorescent signals were still visible after PFA fixation (Suppl. Fig. S2F,G).

### Spatial association of endothelial cells and pericytes

**Figure 2:**
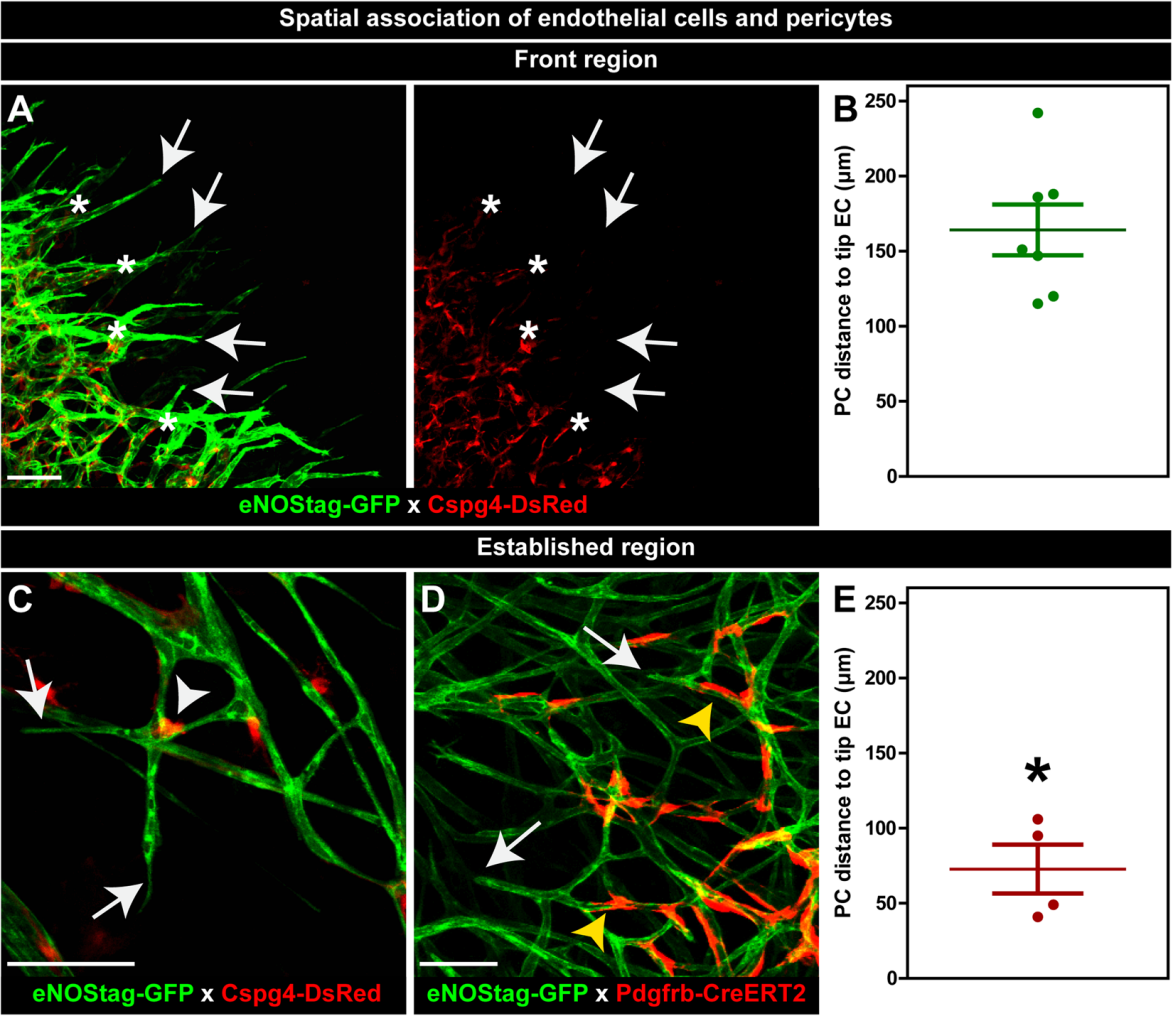
Spatial association of endothelial cells and pericytes. (**A**,**B**) Association in the angiogenic front region. (**A**) Endothelial tip cells (arrow) and pericytes (asterisk) association at the angiogenic front. (**B**) Quantification of pericyte – endothelial tip cell distance in the front region. (**C**–**E**) Association in the established region. (**C**,**D**) Close to the endothelial tip cell (arrow) pericytes are found in the branching points (white arrowhead) or around the tube (yellow arrow). (**E**) Quantification of pericyte – endothelial tip cell distance in the established vessel region. Data represents mean ± SEM. *p < 0.05 versus (**B**). Scale bars represent 100 µm.

Intravital microscopy of above described transgenic animals allows longitudinal spatial evaluation in thick tissue by which the position of endothelial cells and pericytes can be located without intervention. Endothelial tip cells (Fig. 2A; arrow) migrated into a vessel-poor tumor area forming an endothelial sprout. Pericytes (Fig. 2A; asterisk) invaded the non-vascularized area, nicely following the path of endothelial cells, however remained at a specified distance (164.3 ± 16.9 µm) from the endothelial front (Fig. 2B). In the established vasculature pericytes closest to an endothelial tip cell (Fig. 2C; arrow) were found at branching points (Fig. 2C; white arrowhead) or stretched along the endothelial tube (Fig. 2D; yellow arrowhead). Also these pericytes remained at a certain distance (72.9 ± 16.3 µm) from the endothelial tip cell (Fig. 2E). A 3D tissue reconstruction shows the intimate association of Cspg4+ pericytes with an endothelial tube (Suppl. Fig. S3A, Video S2). Pericytes connected to endothelial cells wrapped around the tube even though a single pericyte did not completely envelope the tube (Suppl. Fig. S3Ai, Aii; arrow). This is different from smooth muscle cells in skin tissue, which is observed to be fully wrapped around endothelial tubes (Suppl. Fig. S3B; arrow and Suppl. Video S3).

### Temporal association of endothelial cells and pericytes

**Figure 3:**
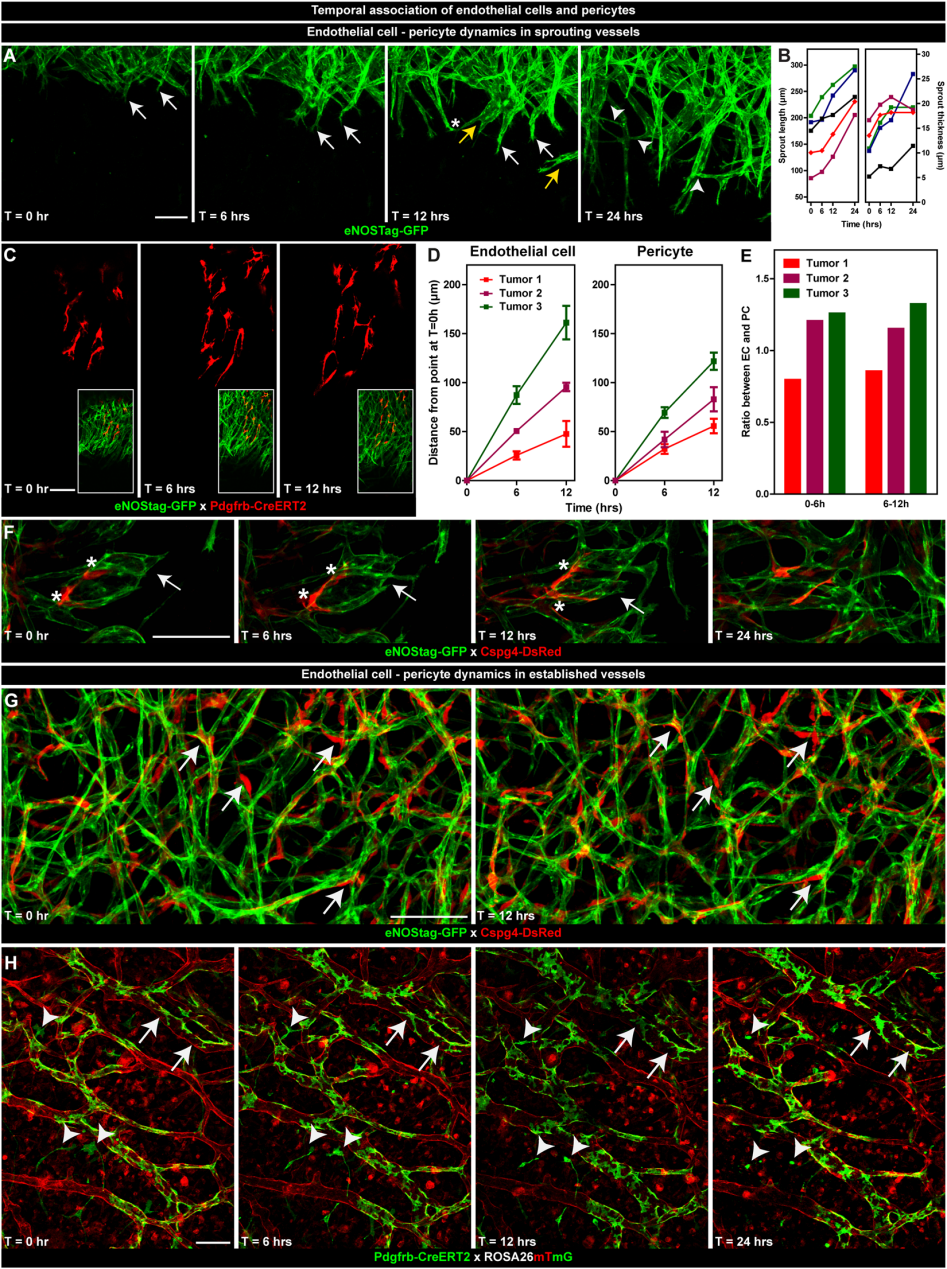
Temporal association of endothelial cells and pericytes. The first evaluation was made when endothelial cells and/or pericytes were clearly seen in the tumor area and re-evaluated 6, 12 and 24 hrs later. (**A**–**F**) Dynamics in sprouting vessels. (**A**) Subsequent projections of endothelial vessel dynamics. Endothelial sprouts invading the unvascularized tumor area (white arrow), splitting of endothelial sprouts (yellow arrow), connections of endothelial sprouts (asterisk), formation of new branches (arrowhead). (**B**) Quantification of sprout length and thickness in time. Each data line represents an individual sprout. (**C**) Subsequential single plane representation of endothelial cell – pericyte association. (**D**,**E**) Quantification of endothelial cell and pericyte migration in 3 individual tumors. Data represent mean ± SD of at least 5 cells. (**F**) Dynamic association of endothelial cells and pericytes in angiogenic vessels indicating endothelial sprouts (arrow) and adjacent pericytes (asterisk). (**G**,**H**) Dynamics in established vessels. (**G**,**H**) Sequential projections of endothelial cells and pericytes in the established vessel region indicating quiescent pericytes (arrow) closely associated with quiescent endothelial cells. (**H**) Pericytes disconnected from the vessel (arrowhead) deteriorating in time. Scale bars represent 100 µm.

Interaction profiles of dynamic processes during angiogenesis are often incomplete or difficult to interpret without temporal information, certainly as several types of specialized cells are involved (for review^29^). Migratory guidance of a tip cell is thought to be driven by stimulatory environmental cues^18,30^ directing the endothelial sprout, which is composed of proliferating stalk cells trailing behind the migrating tip cell. Phalanx endothelial cells^31^ are quiescent cells in the established vasculature and do not show any major change in movement and morphology. We observed a highly interactive cross-talk between endothelial cells as sprouts moved forward (Fig. 3A; white arrow, B), changed diameter (Fig. 3B), separated into new sprouts (Fig. 3A; yellow arrow), connected with other sprouts (Fig. 3A; asterisk), and formed new branches (Fig. 3A; arrowhead). In contradiction, pericytes hardly associated with other pericytes and migrated seemingly independent of each other (Fig. 3C). The distance by which endothelial cells and pericytes migrated forward was variable between tumors (Fig. 3D). However, intratumoral, both cell types maintained their velocity (Table 1) and pericytes followed endothelial cells at almost identical speed (Table 1, Fig. 3E), indicating that angiogenic pericyte behavior (Fig. 3F; asterisk) is most likely controlled by endothelial cells (Fig. 3F; arrow). In established vessels, phalanx endothelial cells were defined as cells that did not show any movement in the evaluation period of 12 or 24 hrs. Pericytes associated with these endothelial cells also showed no significant temporal events (Fig. 3G; arrow). Pdgfrb+ cells detached from a vessel went into apoptosis, as indicated by the granulated cellular debris still expressing GFP, when unable to (re)connect to endothelial cells (Fig. 3H; arrowhead), whereas pericytes that are part of a vessel remained quiescent (Fig. 3H; arrow).

**Table 1:** Velocity of endothelial cells and pericytes.

	Endothelial cells		Pericytes		Ratio EC:PC
	Velocity (nm/s)	Ratio 6 hrs:12 hrs	Velocity (nm/s)	Ratio 6 hrs:12 hrs	
**Tumor 1**					
0–6 hrs	1.19 ± 0.11	1.1	1.49 ± 0.23	1.2	0.8
6–12 hrs	1.10 ± 0.18		1.29 ± 0.17		
**Tumor 2**					
0–6 hrs	2.43 ± 0.06	1.2	1.94 ± 0.34	1.0	1.2
6–12 hrs	2.11 ± 0.01		1.92 ± 0.28		
**Tumor 3**					
0–6 hrs	4.04 ± 0.33	1.1	3.21 ± 0.22	1.1	1.3
6–12 hrs	3.73 ± 0.31		2.82 ± 0.20		

Z-stacks of the same tumor regions were taken of 3 different tumors at 0, 6 and 12 hrs. The distance of individual endothelial cells and pericytes was measured using Fiji and velocity was calculated by dividing the distance of cell traveled between time-point 0 hr and 6 hrs and between 6 hrs and 12 hrs by time. Data represent mean ± SD of at least 5 cells.

### Morphogenetic events of endothelial cells and pericytes

**Figure 4:**
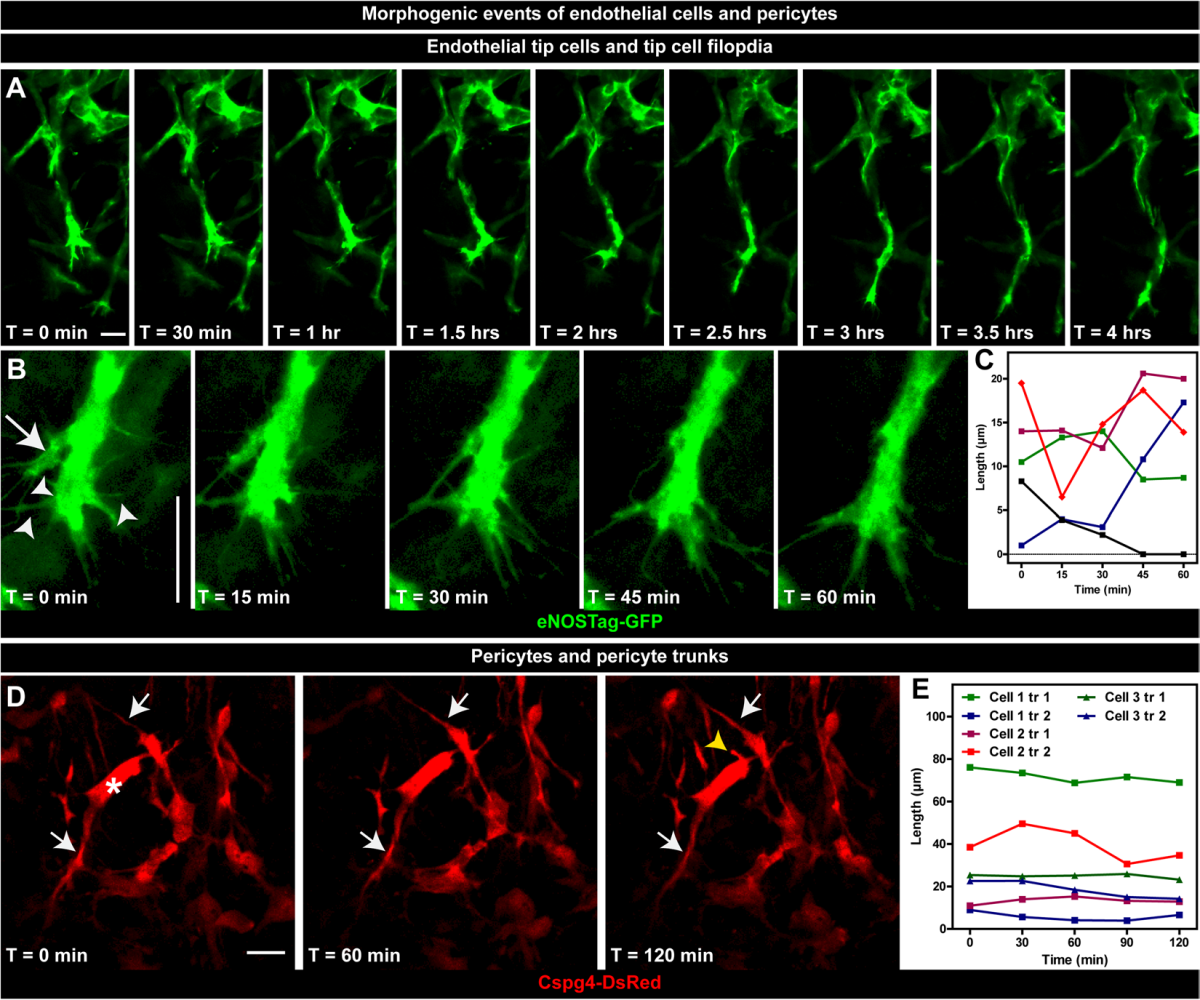
Morphogenetic events of endothelial cells and pericytes. (**A**–**C**) Dynamics of endothelial tip cells. (**A**) Still pictures of a time-lapse movie showing a migrating endothelial tip cell. (**B**) Regressing endothelial cells (arrow) back into the stalk area and dynamics of individual filopodia (arrowhead). (**C**) Quantification of filopodia length in time. Each data line represent an individual filopodium. (**D**,**E**) Sequential images of pericyte dynamics. (**D**) Primary trunks (arrow) protruding from the pericyte cellular body (astriks) and the formation of a new trunk (yellow arrowhead). (**E**) Quantification of the length of pericyte trunks in time. Each data line represent an individual trunk. Scale bars represent 25 µm.

Detailed evaluation of single cells and cellular processes could also be performed using these animals. Continuous imaging of a progressing sprout shows the directionality of endothelial tip cells during migration (Fig. 4A, Suppl. Video S4). We also observed that, during migration, endothelial tip cells, or parts thereof, were regressing back into the stalk area. (Fig. 4B; arrow). Tip cells possessed long and motile filopodia scanning the environment for directional cues and GFP fluorescence was seen in these filopodia (Fig. 4B; arrowhead). In contrast, filopodia could not be visualized using the ROSA-mTmG line. Filopodia of migrating endothelial cells were as dynamic as the tip cell itself and were continuously extending, retracting or even disappearing (Fig. 4C). High-resolution morphogenetic data of pericytes was also collected. Cellular processes, denoted as primary trunks (Fig. 4D; arrow), were seen protruding directly from the cellular body (Fig. 4D; asterisk). Although pericytes were as motile as endothelial cells, as shown in Fig. 3, pericyte trunks did not show the high dynamics of endothelial filopodia (Fig. 4E), what could indicate that these trunks function more for association with endothelial cells rather than migration. Pericytes were highly variable in morphology, which correlated with vessel diameter (Table 2). Pericytes associated with large (>30 µm diameter) vessels (Suppl. Fig. S4A,C; asterisk) had more and shorter primary trunks compared to pericytes associated with vessels of an intermediate (between 30 and 10 µm) diameter and single endothelial cell (<10 µm) vessels. (Suppl. Fig. S4B,C; arrow).

**Table 2:** Correlation between vessel diameter and pericyte morphology.

Vessel diameter (range)	Pericyte trunks		Pericyte branches	
	Number	Length (µm)	Number	Lenght (µm)
>30 µm (74.8–31.9)^#,$^	3.9 ± 0.3^#,$^	12.5 ± 1.4^#,$^	1.0 ± 0.3^#,$^	5.4 ± 0.5
<30> 10 µm (29.1–10.5)	2.7 ± 0.2*	17.2 ± 1.5*	0.4 ± 0.3*	5.7 ± 1.2
<10 µm (8.9–3.0)	2.0 ± 0.2	35.7 ± 4.7	0 ± 0	n.a.

Z-stacks were taken of at least 3 different tumors and vessel diameter, number and length of pericyte trunks and branches measured. ^#^*p* < 0.05 for >30 µm versus <30> 10 µm vessels; ^$^*p* < 0.05 for >30 µm versus <10 µm vessels; *p < 0.05 for <30> 10 µm versus <10 µm vessels. Data represents mean ± SEM of at least 3 cells in 3 different tumors. n.a. = not applicable.

### Retrospective analysis of endothelial cell and pericyte association

**Figure 5:**
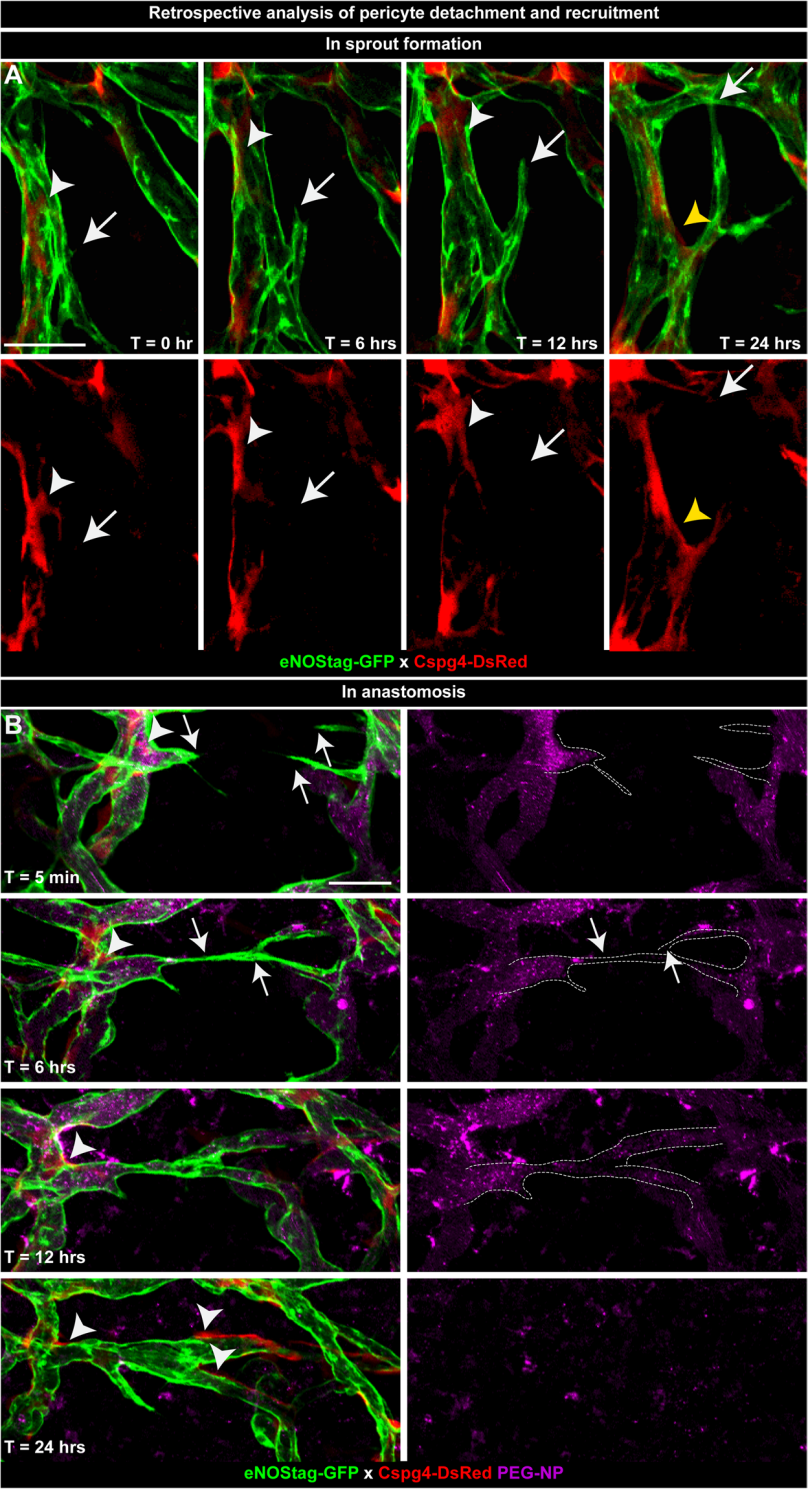
Retrospective analysis of pericyte detachment and recruitment. Images of vessel development taken at T = 0, 6, 12 and 24 hrs were re-evaluated. (**A**) Subsequential projection of a developing endothelial sprout (arrow) and an adjacent dispersing (white arrowhead) and re-emerging (yellow arrowhead) pericyte. (**B**) Forward movement of endothelial cells (arrow) and recruitment of pericytes (arrowhead) during anastomosis. Blood flow is detected using pegylated nanoparticles (PEG-NP) that remain in the blood circulation for approximately 20 hrs. Scale bars represent 50 µm.

As for most dynamic processes it is difficult to predict the onset of an event. However, multi-timed intravital imaging permits retrospective evaluation allowing us to investigate endothelial cell and pericyte dynamics at the start of vessel sprout formation and anastomosis. At positions of emerging sprouts pericytes seemed to be absent at the exact location of endothelial tip cell initiation (Fig. 5A; arrow). Also, pericytes in close vicinity of the beginning endothelial sprout dispersed (Fig. 5A; white arrowhead) and moved away along the existing vessel. More focused experiments need to be done in order to confirm if this is an unrelated cellular movement or a regulated process. Judging from several observations, the latter is more likely as later in the angiogenic process these pericytes re-emerged (Fig. 5A; yellow arrowhead) and associated with stalk endothelial cells of the newly formed sprout. Through injection of a long circulating blood flow marker (Fig. 5B, purple channel) we could evaluate blood flow and movement of endothelial cells (Fig. 5B; arrow) and pericytes (Fig. 5B; arrowhead) during anastomosis. Endothelial cells from opposite vessels moved towards each other and pericytes did not lose contact with endothelial cells and migrated along the vessel tube. Also, blood flow in these endothelial sprouts was present and is known to stimulate regulated forward movement^32,33^.

### Re-evaluating the tissue after whole-mount staining

**Figure 6:**
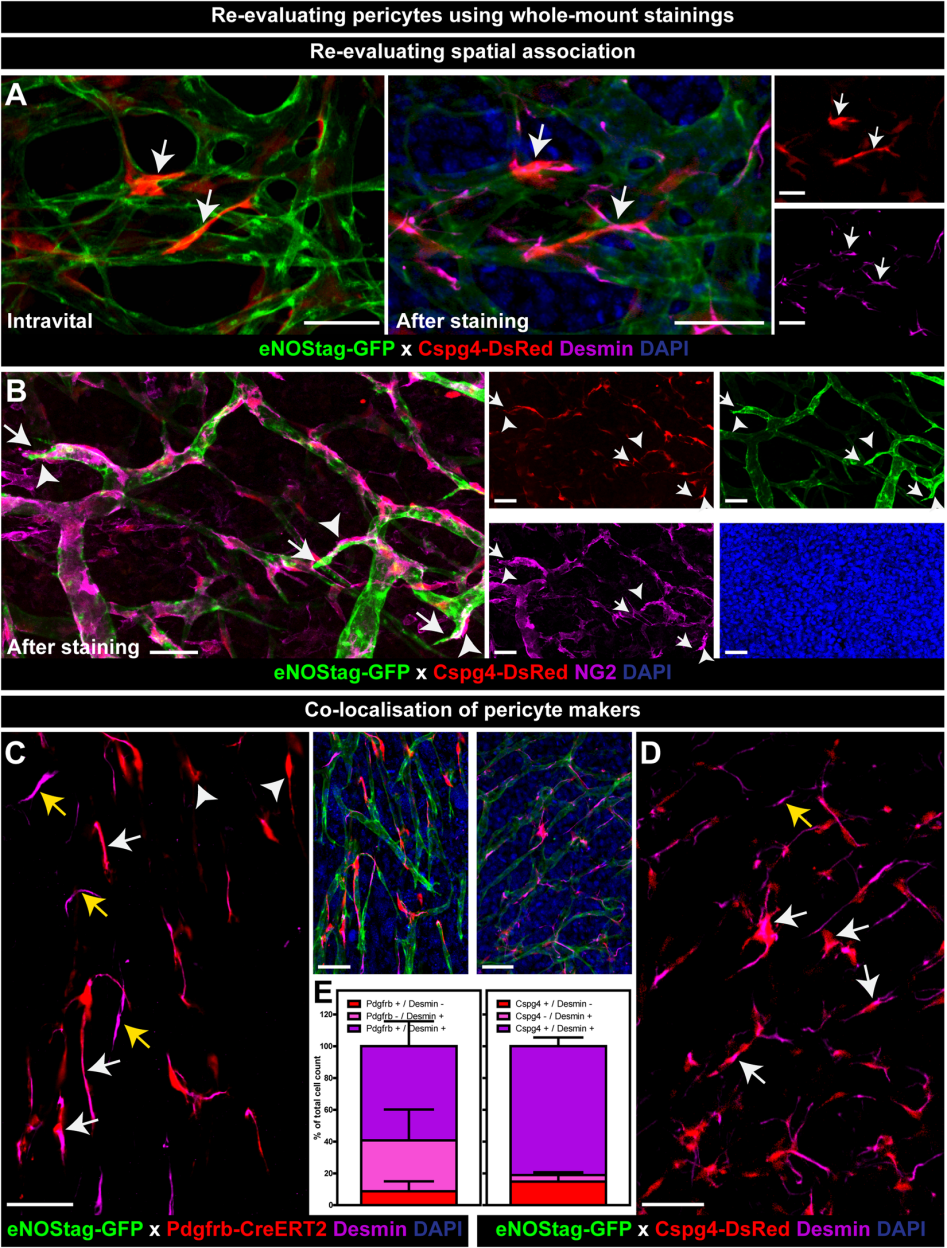
Re-evaluating pericytes using whole-mount staining. (**A**,**B**) Re-evaluating spatial endothelial cell – pericyte association. (**A**) Intravital images of Cspg4+ pericytes shown in Fig. 4 re-evaluated after desmin staining (arrow). (**B**) Cspg4+ pericytes re-stained with NG2 (arrowheads) shown in close proximity of endothelial tip cells (arrow). (**C**–**E**) Co-localization of pericyte markers. (**C**,**D**) Whole-mount staining of tumor tissue showed Pdgfrb+ or Cspg4+ pericytes co-expressing desmin (white arrow), Pdgfrb+ pericytes not stained for desmin (arrowhead), and Pdgfrb- or Cspg4- pericytes stained positive for desmin (yellow arrow). (**E**) Quantification of co-expression of Pdgfrb or Cspg4 with desmin. Data represent mean ± SEM. Scale bars represent 50 µm.

There is still much unknown about pericyte origin and several subpopulations are believed to exist^16,34,35^. Staining pericytes with different markers will be helpful in identify these subgroups. To re-evaluate endothelial cell – pericyte association and investigate pericyte identity, tumor tissue was stained as a whole-mount after intravital evaluation. A number of antibodies for pericytes were tested: desmin (Fig. 6A, Suppl. Fig. S5A), NG2 (Fig. 6B), Pdgfrb (Suppl. Fig. S5B,D) and SMA (Suppl. Fig. S5C) and were found to stain pericytes in the dissected tumor. GFP, DsRed and mTomato fluorescence were still visible after fixation, bypassing the need for counterstaining. Changes in cellular morphology after fixation were minimal and intravitally evaluated regions were found back after staining allowing quantification of pericyte subpopulations in relation to their cellular morphology and vessel position (Fig. 6A; arrow). B16BL6 and LLC tumors are surrounded by fibrous encapsulating tissue and Pdgfrb (Suppl. Fig. 5B; asterisk) and SMA antibodies interacted with this tissue limiting their use for Z-stack imaging. Desmin, an intracellular intermediate filament protein and seen as fibers throughout the cell, worked best for whole-mount staining, as it penetrated tissue better and gave less background. Using whole-mount staining the position of an endothelial tip cell (Fig. 6B, Suppl. Fig. S5A; arrow) and nearby pericyte was not compromised (Fig. 6B, Suppl. Fig. S5A; arrowhead). Of all stained cells 59.2 ± 15.7 percent were Pdgfrb/desmin double positive (Fig. 6C; white arrow, Fig. S6) and 81.1 ± 5.5 percent were Cspg4/desmin double positive (Fig. 6D; white arrow, Fig. 6E), indicating that the majority of endogenously stained pericytes also stained positive for a second pericyte marker.

## Discussion

Several pre-clinical models are available to study angiogenesis *in vivo*: mouse embryos, retina, bone and tumor, chick corneal micropocket assay and zebrafish (for review^36–38^). Embryo’s, retina’s, bones and sectioned tumors are relative easy techniques and offer rapid, quantitative but static analysis. Chick corneal micropocket assay and zebrafish permit non-invasive observation and long-term monitoring. However these models represent non-mammalian embryonic angiogenesis and the possibility for additional staining is minimal. Intravital window models (for review^39,40^) are often used to investigate tumor pathophysiology and therapeutic effects^41–43^. Although tumor angiogenesis is chaotic and less controlled it features all stages of vessel development and is closer related to the human setting compared to chick and zebrafish models. Therefore, to study a complex biological system like angiogenesis, it is important to combine several models complementing their advantages^44–46^. In this report we demonstrate the use of several transgenic mouse lines using a developing tumor as an angiogenic model showing the dynamic interaction between endothelial cells and pericytes in 4D high resolution followed by whole-mount re-evaluation. Cross-breeding Cspg4-DsRed, as well as Pdgfrb-CreERT2 mice, with eNOStag-GFP animals produced offspring in which endothelial cells and pericytes were clearly distinguishable from each other. GFP was expressed in the Golgi, membrane and filopodia of endothelial cells allowing visualization of ingrowth of vessel sprouts, interactions between sprouts, progression or regression of endothelial tip cells and dynamics of individual filopodia. With the exception of visualizing endothelial filopodia, Pdgfrb-CreERT2 x ROS-mTmG can also be used although the membrane-bound eGFP gives us a less accurate representation of pericyte morphology. Cytosolic DsRed and tdTomato fluoresce throughout the pericyte body and processes, allowing visualization of morphological characteristics of pericytes. Although endogenous labeling of pericytes did not permit single cells perception, their more dispersed distribution made individual cell tracking possible. In these described models, which also allow retrospective analysis, endothelial cells and pericytes were studied in the correct spatiotemporal context. Appearance of an endothelial tip cell is a crucial cellular event in sprouting angiogenesis and there is a delicate feedback loop in the signaling pathway notch/DLL4/Jagged-1 determining which cell becomes the tip cell, and which cells form the new sprout; the stalk cells^47,48^. However the dynamic nature in tip cell replacement, tip/stalk cell switch and the exact involvement of directional cues are still not clear^30,45,46,49–51^. Simultaneously, pericytes migrate, associate and disperse alongside an endothelial sprout, although whether or not pericytes guide these endothelial sprouts needs further investigation^51–53^. Sectioning of tissue, required for histology, generates blind ended vessels which can wrongly be interpreted as endothelial tip cells. Although whole-mount staining procedure needs intensive validation and, this is especially true for the induced Cre-line, endogenous labeling was not 100% of all cells, the spatial position of endothelial cells and pericytes are not compromised. Therefore, this model will also help investigating of signaling pathways^54^ in the dependency of pericytes on endothelial cells to migrate.

Taken together, the mouse lines presented here, in combination with high resolution 4D imaging and whole-mount histology, will substantially contribute to the investigation of spatiotemporal association and molecular pathways of endothelial cells and pericytes during angiogenesis.

## Methods

### Animal work and breeding history

Animal experiments were approved by the Instantie voor Dierenwelzijn Erasmus MC and MPI institutional animal ethics committee and conducted with permissions granted by the Nederlandse Dierexperimentencommissie and by the Landesamt für Natur, Umwelt und Verbraucherschutz (LANUV) of North Rhine-Westphalia, Deutschland. The experiments were performed according to the European directive 2010/63/eu on the protection of animals used for scientific purposes. ROSA reporter lines and eNOStag-GFP were bred homozygous. Cspg4-DsRed was kept hemizygous as recommended by the developer and the Pdgfrb(BAC)-CreERT2 line to avoid overexpression. Male Cre drivers were crossed with a female reporter or female eNOStag-GFP. All breeding combinations produced in general a standard number of offspring and male/female ratio. No harmful phenotype was observed in breeding animals and their experimental offspring. Also Cre-induction using tamoxifen showed no toxicity.

### Generation of the animals

The eNOStag-GFP mouse is a variation of the eNOS-GFP line, which has an elevated eNOS level^55^. In the tag line, a truncated eNOS, maintaining the localization pattern however losing the ability to overproduce eNOS, is fused to GFP (Suppl. Fig. S1a). To ensure protein expression, a DNA fragment containing a 6.3 kb promoter sequence and 123 bp of the first human eNOS gene exon were isolated from a home-made human cosmid library and fused in frame with GFP cDNA. This DNA was injected into fertilized FVB/n donor oocytes and transplanted into oviducts of pseudopregnant B16xCBA mice. A cDNA encoding tamoxifen-inducible Cre recombinase (Suppl. Fig. S1b) followed by a polyadenylation signal sequence and a FRT-flanked ampicillin resistance cassette were introduced by recombination of the start codon of Pdgfrb in the bacterial artificial chromosome clone RP24-62H17TJ (BACPAC Resources Center, Children’s Hospital Oakland Research Institute). After Flp-mediated excision of the ampicillin resistance cassette in bacteria, the resulting constructs were validated by PCR analysis and used in circular form for pronuclear injection into fertilized mouse oocytes^56^.

The genotype of eNOStag-GFP mice was determined by polymerase chain reaction using the following primers; GFP-Forward, 5-AGCTGACCCTGAAGTTCATCTG-3; GFP-Revers, 5-GACGTTGTGGCTGTTGTAGTTG-3. The reaction was performed on toe or ear DNA for 35 cycles at 30 seconds at 94 °C, 60 seconds at 65 °C and 60 seconds at 72 °C and produced a product of 300 bp. For the genotyping of the Cre-line the following primers were used; Cre-Forward, 5-GTGGCAGATGGCGCGGCAACACCATT-3; Cre-Revers, 5-GCCTGCATTACCGGTCGATGCAACGA-3. The thermal profile was 30 seconds at 94 °C, 72 seconds at 65 °C and 60 seconds at 72 °C for 35 cycles producing a product of 720 bp. Cspg4-DsRed (stock 008241), ROSA-mtdTomato-meGFP (ROSA-mTmG; stock 007676), ROSA-tdTomato (stock 007914) mice were purchased from Jackson laboratory. To create experimental offspring, eNOStag-GFP animals were crossed with Cspg4-DsRed mice (Suppl. Fig. S1c) and Pdgfrb(BAC)-CreERT2 with ROSA-mTmG (Suppl. Fig. S1d). For triple transgenic mice a Pdgfrb(BAC)-CreERT2 x ROSA-tdTomato line was first created after which the males were crosses with eNOStag-GFP females (Suppl. Fig. S1e). All animals were kept on a C57bl/6 background.

### Cre Induction

Tamoxifen (Sigma Aldrich) was dissolved in ethanol/peanut oil (1/3) mixture at a concentration of 10 mg/ml. This was further diluted 1/10 in peanut oil and 50 µl was injected into the pup at postnatal day 1 and 2. Animals were allowed to age for dorsal skinfold transplantation (Suppl. Fig. S1f).

### Dorsal skinfold transplantation

Murine melanoma B16BL6 and Lewis lung carcinoma (LLC) cells were maintained in DMEM medium and 10% heat-inactivated fetal calf serum. Subcutaneous tumors were generated by injecting 1 × 10^6^ tumor cells in the flank of a wildtype animal under isoflurane inhalation anesthesia. This tumor was used for transplantation into the dorsal skinfold. Preparation of tumor implantation in the dorsal skinfold chamber is previously described^41,57^ and performed on mice of 10 weeks old and at least 20 g (Suppl. Fig. S1F). Briefly, mice were anesthetized using isoflurane inhalation anesthesia and hair was removed from the back of the animal. After dissecting the skin, leaving fascia and opposing skin intact, the skinfold of the mouse was sandwiched between two frames, fixed with two light metal bolts and sutures. A small piece of tumor (0.1 mm^3^), obtained from the wildtype bulk animal, was transplanted in the fascia. The backside was closed with a 10 mm filler glass and standard 12 mm diameter microscopic glass and the front with a 12 mm photoetched grid glass (Electron Microscopy Science, USA). The glasses are held in place with a thin metal ring. The mice were individually housed in an incubation room with an ambient temperature of 30 °C and a humidity of 70%.

### Intravital evaluation

The ingrowth of tumor vessels into B16BL6 and LLC tumors started around 7 days after implantation and took 10 to 13 days to form a complete functional vasculature. For intravital confocal microscopy, mice were anesthetized using isoflurane inhalation anesthesia and placed on a heated stage of a Leica SP5 multiphoton microscope (Leica Microsystems)^57^. As tumors were obtained from a wildtype animal all fluorescent cells were indicative to the host. To perceive blood flow, animals were injected with pegylated nanoparticles labeled with a far-red fluorescent marker^58^ which stays in the blood circulation for approximately 20 hrs and/or with 30 µl Hoechst 33342 (Sigma Aldrich; 10 mg/ml), a small molecule that is taken up by living cells.

### Whole-mount staining

After intravital evaluation, the glass of the window was carefully removed and the tumor tissue was stained as a whole-mount allowing extra identification of intravitally evaluated pericytes. The tumor was fixed in 4% paraformaldehyde (PFA), permeabilized, stained with rabbit anti desmin (Abcam), guinea pig anti NG2 (kindly donated by Prof. Stallcup), rat anti Pdgfrb (eBioscience) or mouse anti SMA-Cy3 (Sigma Aldrich) and, dependent on the already endogenous fluorescence, counterstained with a corresponding secondary Alexa Fluor 561 or 643 antibody (Molecular Probes) and DAPI (Sigma Aldrich). The tissue was mounted using glycerol/gelatin (Sigma Aldrich) in CoverWell imaging chambers (Sigma Aldrich) and evaluated using a Leica SP5 multiphoton microscope.

### Imaging and representation

Imaging, intravital^57^ as well as histological, was performed using a Leica SP5 multiphoton microscope. For intravital microscopy, an APO 10.0 (NA 0.4) or APO 20.0 (NA 0.5) dry objective lens and, for histology, an APO 10.0 (NA 0.4) dry, APO 20.0 (NA 0.7) dry or an APO 40.0 (NA1.25–0.75) oil lens were used. Fluorescence was excited with a 405 nm diode, 488 nm argon, 543 nm and 633 nm helium-neon laser. The reflection of the grid etched in the cover glass was used as a reference point for multi-time imaging. Unless mentioned otherwise, images are presented in the results section as maximal projection of a single tile Z-stack.

### Analysis of pericyte morphology

Determining pericyte morphology was performed by making 3D reconstruction from high-resolution confocal stacks using Amira software. The morphological characteristics of individual pericytes is based on a report by Hartmann *et al*.^13^ in which pericytes are morphologically evaluated in brain tissue as this corresponds to morphological features seen in the tumor. The pericyte cellular body was identified by the presence of the nuclei visualized by Hoechst or DAPI, primary trunks as cellular processes protruding directly from the cellular body and primary branches as thin singular stands splitting from the primary trunk. Care was taken to only analyze isolated pericytes and processes longer than 3 µm were counted.

### Data analysis

Fiji (Wayne Rasband, NIH) was used for data analysis. Dynamic vessel sprout length was established by measuring the length between tip of the vessel and first branching point. Subcellular length of endothelial filopodia and pericyte processes was established by measuring the length between tip of the filopodia/process and center of the cellular body. Images taken at time-point 0, 6, 12 and 24 hrs were used to measure migration distance and velocity of individual cells. At time-point 0 hr cells were selected and traced in the following images. Velocity was calculated by dividing the recorded distance by time. To determine co-expression of pericyte markers, co-labeled cells were traced throughout the channels of the individual images of the Z-stack, using the nuclear staining to identify single cells and were counted manually.

### Statistics

Results were evaluated for statistical significance with the Mann Whitney U test using IBM SPSS Statistics 21. P-values below 0.05 were considered statistically significant. Data was obtained from at least 3 individual experiments.

## Electronic supplementary material

Supplemental Video S1

Supplemental Video S2

Supplemental Video S3

Supplemental Video S4

Supplemental info
