# Supplementary material for: Spatiotemporal endothelial cell – pericyte association in tumors as shown by high resolution 4D intravital imaging
Source: Sci Rep. 2018 Jun 25;8:9596. doi: 10.1038/s41598-018-27943-8 (PMC6018425; doi:10.1038/s41598-018-27943-8)
Supplement: Supplementary file 5 — Supplemental info [file 41598_2018_27943_MOESM5_ESM.pdf]

Supplementary Methods and Figures

Spatiotemporal endothelial cell – pericyte association in tumors as shown by high resolution 4D intravital imaging. Ann LB. Seynhaeve, Douwe Oostinga, Rien van Haperen, Hanna M. Eilken, Susanne Adams, Ralf H. Adams & Timo LM. ten Hagen

1.Supplementary Methods

Assessment of retina, tumor and skin

Retina dissection is previously described[1]. Briefly, after the animal was killed, eyes were isolated and fixed for 30 min at room temperature with 4% PFA. The cornea and iris, outer layer of the eye, sclera and pigmented retina layer were removed. After dissection of the hyaloid vessels from the inside of the eye, four incisions in the retina were made for flat-mounting on a microscope glass using fluoromount-G (Southern Biotech).

In addition to whole-mount stainings, stainings were also performed on 100 µm thick vibratome section. Subcutaneous tumors were generated by injecting $1x10^6$ tumor cells in the flank of an animal under isoflurane inhalation anesthesia and allowed to developed to a size of approximately 300 mm^3. The animal was killed, tumor dissected, fixed for 24 hrs at 4ºC with 4% PFA and embedded in 4% low-melting agarose (Serva Electrophoresis GmbH). Sections of 100 µm were made using a Leica vibratome, permeabilized, stained with rabbit anti ß-COP (Abcam) or rabbit anti FAP-1 (Sigma Aldrich) and counterstained with Alexa Fluor donkey anti rabbit 643 secundary antibody (Molecular Probes) and DAPI (Sigma Aldrich). The tumor was mounted using fluoromount-G. TUNEL staining was performed with the Click-iT Plus TUNEL assay (Molecular probes) according the manufacture's instruction with three-times longer wash and incubation steps to allow deeper penetration. Tumor sections were mounted using fluoromount-G/DAPI.

A square of skin was dissected from the back of the animal, fixed overnight at 4ºC with 4% PFA, washed and mounted using glycerol/gelatin (Sigma Aldrich) in CoverWell imaging chambers (Sigma Aldrich). Evaluation was performed using a Leica SP5 multiphoton microscope.

2.Inventory

Suppl. Fig. S1: related to Methods and Fig.1

Suppl. Video S1: related to Fig.1

Suppl. Fig. S2: related to Fig.1

Suppl. Fig. S3: related to Fig.2

Suppl. Video S2: related to Suppl. Fig. S3

Suppl. Video S3: related to Suppl. Fig. S3

Suppl. Video S4: related to Fig.4

Suppl. Fig. S4: related to Fig.4

Suppl. Fig. S5: related to Fig.6

3.Supplementary figures

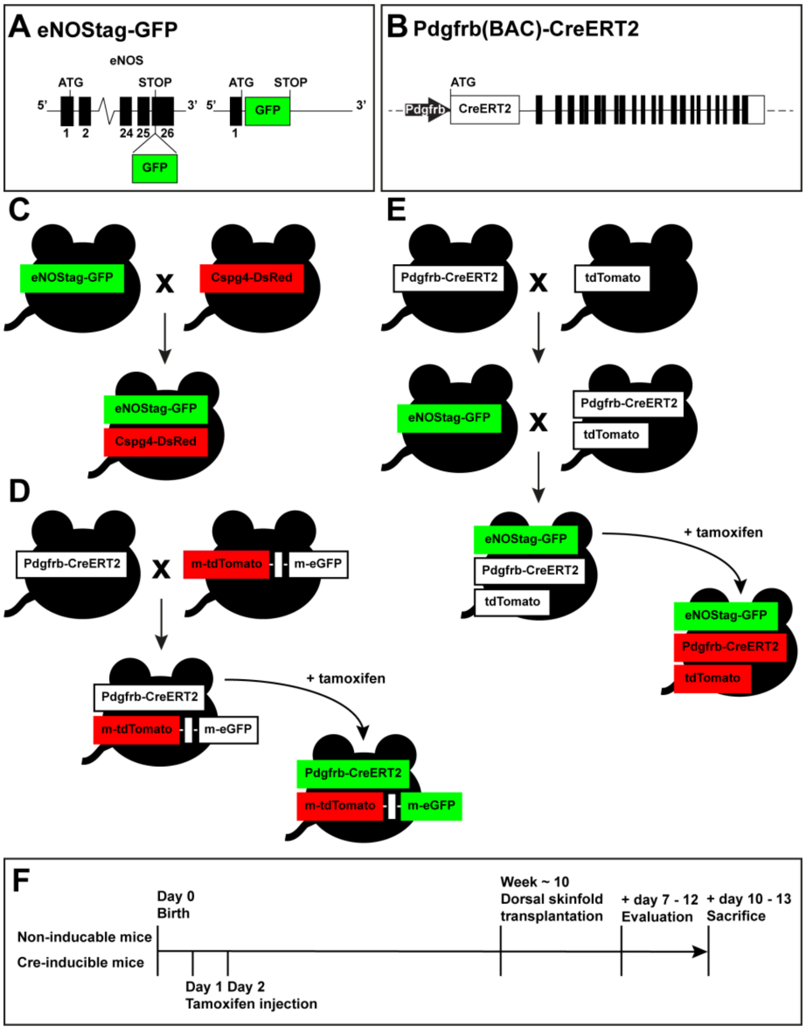

Supplementary Figure S1. Animal generation and procedure. (A,B) Schematic representation of eNOStag-GFP (A) and Pdgfrb(BAC)-CreERT2 (B) gene. (C-E) Breeding schedule to generate eNOStag-GFP x Cspg4-DsRed (C), Pdgfrb-CreERT2 x ROSA-mtdTomato-meGFP (D) and eNOStag-GFP x Pdgfrb-CreERT2 x ROSA-tdTomato (E) experimental offspring. (F) Animal procedure for non-inducible and cre-inducible experimental animals.

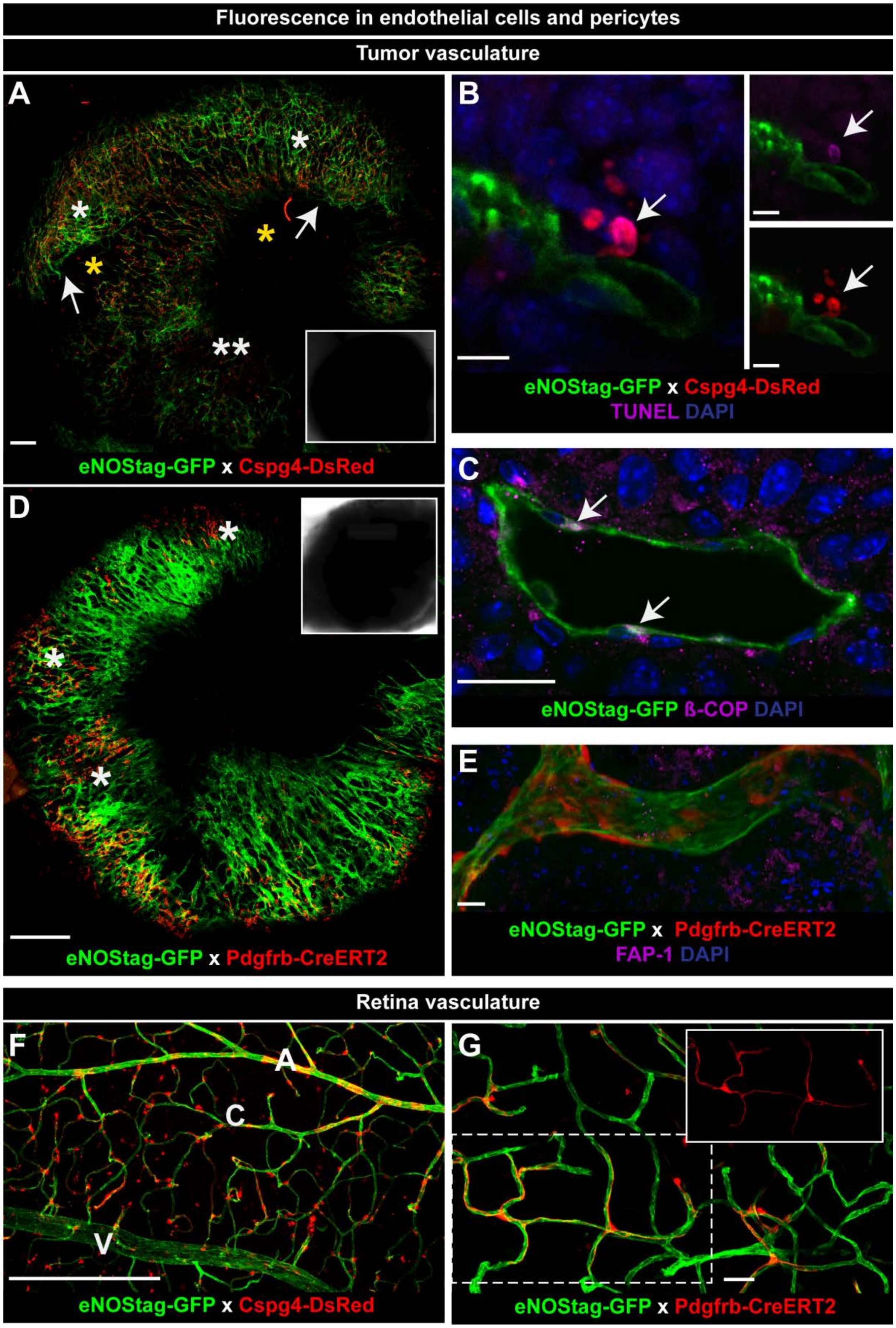

Supplementary Figure S2. Fluorescence in endothelial cells and pericytes. (A-E) Tumor distribution of endothelial cells and pericytes. (A) Homogenous fluorescence distribution of GFP in endothelial cells and DsRed in Cspg4+ pericytes. Intravital image is a maximal projections of a tile scan taken from a B16BL6 tumor. Scale bar represents 500 µm. Insert represents low resolution bright field. (B) TUNEL and DAPI staining in a sectioned tumor from an eNOStag-GFP x Cspg4-DsRed mice. An apoptotic Cspg4+ cells (arrow) is identified by granulated cell debris still expressing DsRed which co-stains for DNA fragmentation. Scale bar represents 25 µm. (C) ß-COP and DAPI staining of a sectioned tumor from an eNOStag-GFP mouse. Endogenous GFP expression shows co-localization with the golgi staining (arrow). Image is a single plane image. Scale bar represents 25 µm. (D) Fluorescence distribution of tdTomato in Pdgfrb+ pericytes in hotspots (asterisk). Intravital image is a maximal projections of a tile scan taken from a

B16BL6 tumor. Scale bar represents 500 µm. Insert represents low resolution bright field. (E) FAP-1 fibroblast staining of a sectioned tumor from an eNOStag-GFP x Pdgfrb(BAC)-CreERT2-TdTomato mouse. Pdgfrb+ cells are FAP-1 negative. Scale bar represents 25 µm. (F,G) Endothelial cell – pericyte association in retina. (F) GFP and DsRed fluorescence in retina dissected from an adult eNOStag-GFP x Cspg4-DsRed mouse. Recognizable are the artery (A), vein (V) and capillaries (C). Scale bar represents 250 µm. (G) GFP and tdTomato fluorescence in capillaries of the retina dissected from an adult eNOStag-GFP x Pdgfrb(BAC)-CreERT2-TdTomato mouse. Scale bars represent 25 µm.

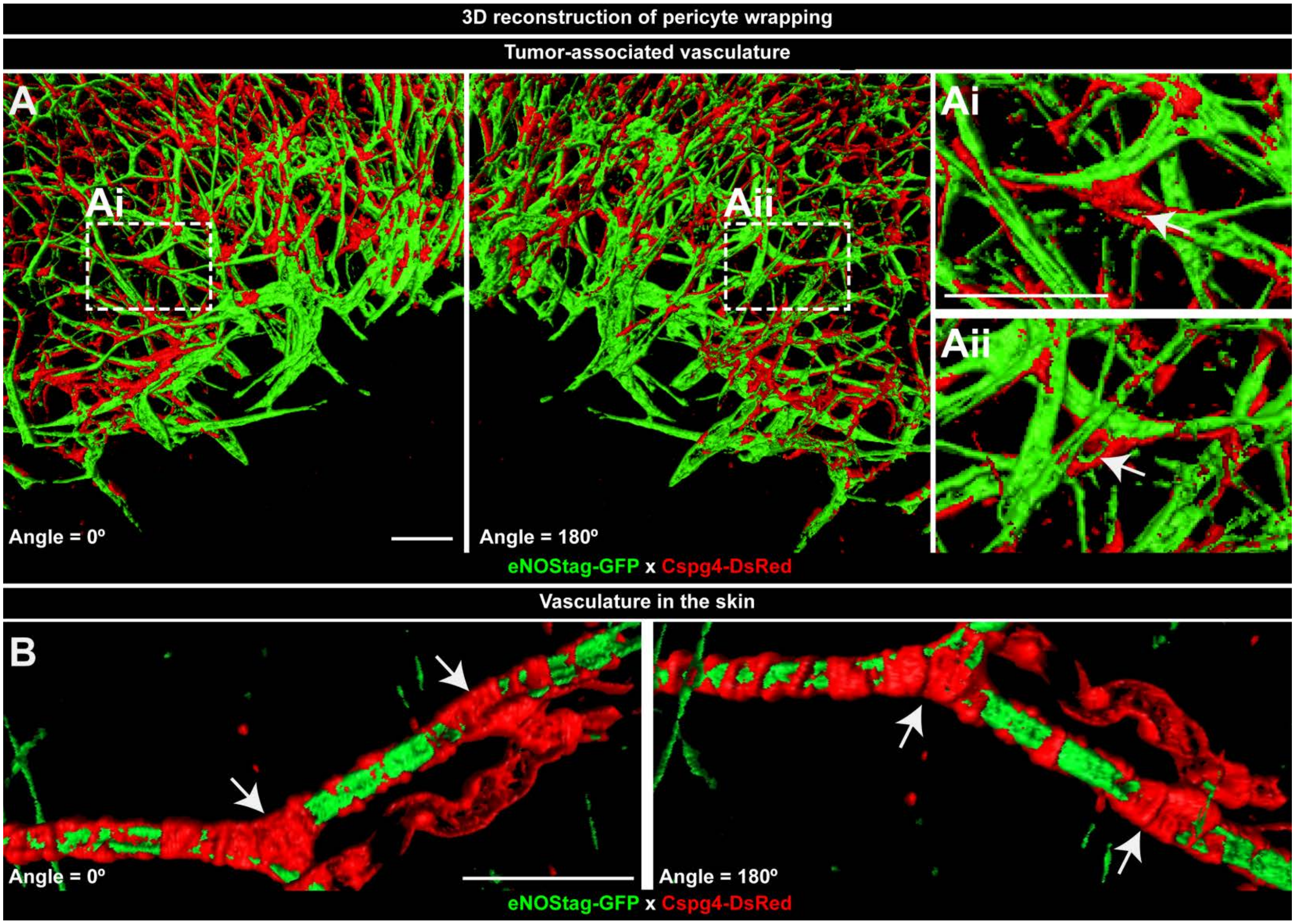

Supplementary Figure S3. 3D reconstruction of perivascular cell wrapping in the vasculature of (A) tumor and (B) skin. (A) Still pictures at the 0º and 180º angle of the tumor-associated vasculature. (Ai, Aii) A pericyte wrapped around the tube does not completely cover the endothelial space (arrow). (B) Still pictures at the 0º and 180º angle of the vasculature in the skin. Smooth muscle cells wrapped several times around the endothelial tube (arrow). Scale bars represent 100 µm.

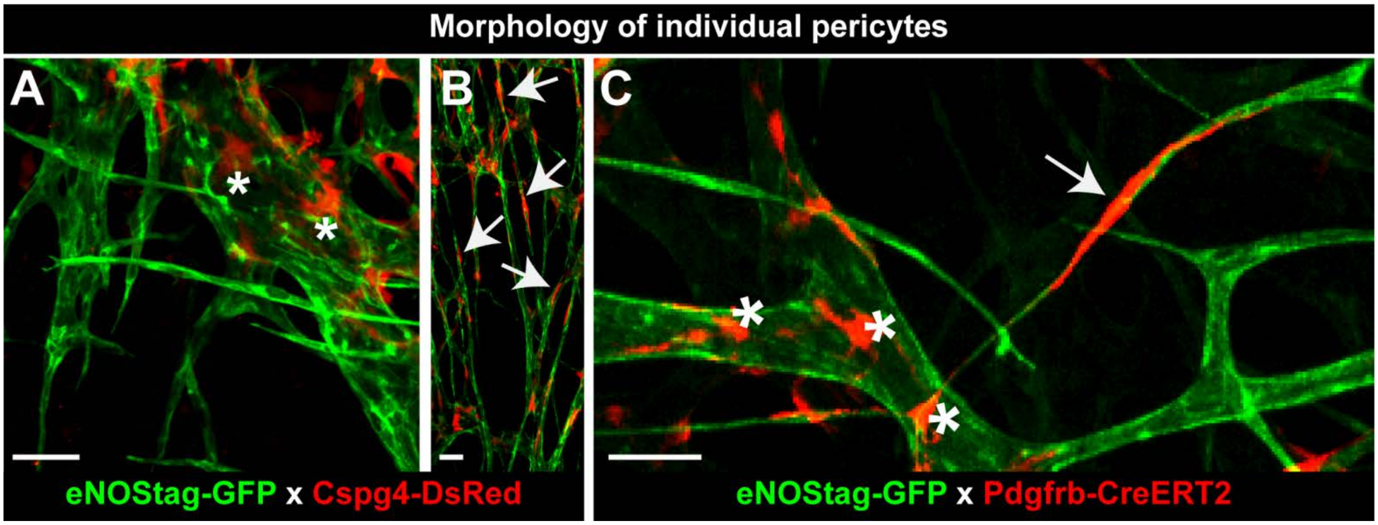

Supplementary Figure S4. Morphology of individual pericytes. (A-C) Relationship between pericyte morphological characteristics and vessels. (A) Cspg4+ pericytes covering a tick vessel have a round cellular body (asterisk). (B) Stretched Cspg4+ pericytes covering thin stretched endothelial tubes (arrow). (C) Pdgfrb+ pericytes covering a thick vessel (asterisk) and a Pdgfrb+ pericyte aligned with a stretched endothelial tube (arrow). Scale bars represent 50 µm.

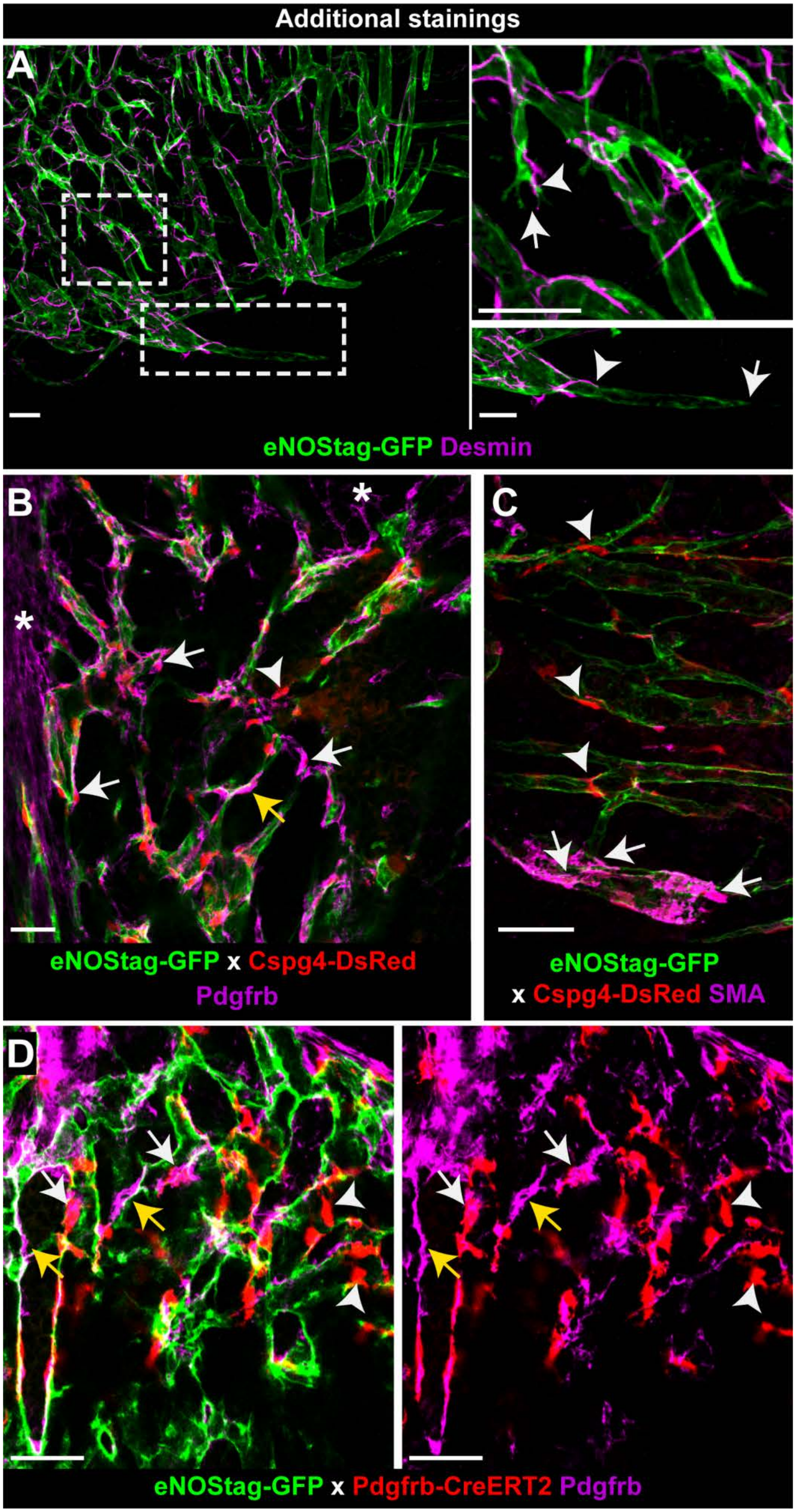

Supplementary Figure S5. Additional whole-mount stainings. (A-D) Staining using antibodies of the dissected tumor tissue after intravital evaluation. (A) Desmin stained pericyte (arrowhead) associated with an endothelial tip cell (arrow). (B-D) A majority of endogenous Cspg4+ or Pdgfrb+ labeled cells also stain positive when using a Pdgfrb (B,D) or SMA (C) (white arrow) antibody. Cells that are not stained for the antibody (yellow arrow) or cells that are only stained with the antibody (white arrowhead) are also observed. Tumors are encapsulated with fibrous tissue which stains positive for Pdgfrb (asterisk). Image B and D represent a maximal projection of 20 µm. Scale bars represent 50 µm.

4.Legends related to supplementary videos

Supplementary Video S1. Video representation of a Z-stack in the red channel of the ROSA mTmG. Z-stack is taken of a B16BL6 tumor in the ROSA mTmG mouse. Cells in the bloodstream express mTomato and are seen in the blood flow. Frame rate is 3 fps. Scale bar represents 100 µm.

Supplementary Video S2. 3D reconstruction of pericytes wrapping in tumor-associated vasculature.
Supplementary Video S3. 3D reconstruction of smooth muscle cells wrapping in skin vasculature.

Supplementary Video S4. Progressing endothelial tip cells. Forward movement of an endothelial sprout was achieved by making a time-lapse movie. Every 10 min a Z-stack was taken during 4 hrs. Frame rate is 3 fps.

5.References

1. Pitulescu, M.E., Schmidt, I., Benedito, R. & Adams, R.H. Inducible gene targeting in the neonatal vasculature and analysis of retinal angiogenesis in mice. Nat Protoc 5, 1518-1534 (2010).
